# Immune-Inflammatory Response in Lifespan—What Role Does It Play in Extreme Longevity? A Sicilian Semi- and Supercentenarians Study

**DOI:** 10.3390/biology13121010

**Published:** 2024-12-04

**Authors:** Giulia Accardi, Anna Calabrò, Rosalia Caldarella, Calogero Caruso, Marcello Ciaccio, Marta Di Simone, Mattia Emanuela Ligotti, Serena Meraviglia, Rosa Zarcone, Giuseppina Candore, Anna Aiello

**Affiliations:** 1 Laboratory of Immunopathology and Immunosenescence, Department of Biomedicine, Neuroscience and Advanced Diagnostic, University of Palermo, 90134 Palermo, Italy; giulia.accardi@unipa.it (G.A.); anna.calabro@unipa.it (A.C.); rosa.zarcone@community.unipa.it (R.Z.); anna.aiello@unipa.it (A.A.); 2 Department of Laboratory Medicine, University Hospital “P. Giaccone”, 90127 Palermo, Italy; rosalia.caldarella@policlinico.pa.it (R.C.); marcello.ciaccio@unipa.it (M.C.); 3 Section of Clinical Biochemistry, Clinical Molecular Medicine and Clinical Laboratory Medicine Department of Biomedicine, Neurosciences and Advanced Diagnostics, University of Palermo, 90127 Palermo, Italy; 4 Central Laboratory of Advanced Diagnosis and Biomedical Research, University Hospital “P. Giaccone”, 90127 Palermo, Italy; martadesimone@unipa.it (M.D.S.); serena.meraviglia@unipa.it (S.M.); 5 Department of Biomedicine, Neurosciences and Advanced Diagnostics, University of Palermo, 90127 Palermo, Italy; 6 ISMETT-IRCCS Mediterranean Institute for Transplants and Highly Specialised Therapies, 90127 Palermo, Italy; mligotti@ismett.edu

**Keywords:** aging, ARIP, biological aging, immune-inflammatory response, inflamm-aging, INFLA-score, longevity, semi-supercentenarians, SIRI

## Abstract

Studying models of healthy aging and exceptional longevity is crucial to understanding a possible longevity signature, as most show resistance to age-related diseases. In particular, semi- and supercentenarians are a highly selected group, having survived significant adversities, including the Spanish flu and COVID-19 pandemics, indicating distinctive immune system characteristics. This paper analyzes the inflammatory scores (INFLA-score, Systemic Inflammation Response Index (SIRI)) and Aging-Related Immune Phenotype (ARIP) indicators calculated from the dataset of the DESIGN project, including 249 participants aged 19–111 years, aiming to understand the immune-inflammatory (IMFLAM) role in achieving longevity. Statistical analyses, including linear regression and one-way ANOVA, were performed to explore the correlations between these parameters and age. Both INFLA-score and SIRI showed a significant increase with age. However, no statistical differences were found when comparing the values of semi- and supercentenarians to other age groups, which are similar to adults and lower than younger centenarians. Regarding ARIP values, it is noteworthy that when comparing the CD8^+^ Naïve/Effector scores between groups, no significant differences were observed between the semi- and supercentenarian group and the other groups. These results support the idea that the control of IMFLAM response can promote extreme longevity.

## Simple Summary

By analyzing inflammatory scores (INFLA-score, Systemic Inflammation Response Index—SIRI) and the Aging-Related Immune Phenotype (ARIP) in 249 participants aged 19–111 years, this study investigates the role of immune-inflammatory responses in semi- and supercentenarians who have survived significant challenges like pandemics. Statistical analyses indicated that the INFLA-score and SIRI increase with age, but no significant differences were observed between semi- and supercentenarians and other age groups. Moreover, ARIP values, specifically CD8 Naïve/Effector scores, calculated from a subcohort of 54 individuals, showed no notable differences across groups. These findings suggest that effective management of immune-inflammatory responses may play a role in achieving extreme longevity.

## 1. Introduction

Positive biology focuses on understanding the biological mechanisms that promote health and longevity. Rather than studying diseases and their causes, positive biology seeks to identify the biological factors that contribute to human resilience and optimal functioning, including relevant biomarkers and physiological processes. This approach is especially valuable in an aging world, which faces a growing set of public health challenges [1,2].

In this context, examining models of healthy aging and exceptional longevity may give us a key lesson. Among Long-Lived Individuals (LLIs, ≥90 years), centenarians (≥100 years), including semi-supercentenarians (105–109 years) and supercentenarians (≥110 years), are a focus of extensive research. Most of them are resistant to or manage age-related diseases such as cancer, diabetes, cardiovascular diseases, and stroke. Thus, they are categorized as survivors, escapers, or delayers [1,3].

However, it is important to recognize that the growing number of centenarians is due to advancements in hygiene and sanitation, as well as healthier lifestyles. Consequently, contemporary and future centenarians are likely to be less selectively unique than those from previous decades [4].

Semi- and supercentenarians represent a uniquely selective group. They have endured significant adversities, including two World Wars, the Spanish flu, and the COVID-19 pandemics [5,6,7,8,9]. Therefore, it is plausible to infer that their immune systems exhibit remarkable traits that can provide insights into the mechanisms influencing the achievement of such extreme longevity [10,11].

Age-related dysregulation of immune-inflammatory responses (IMFLAM) has indeed been recognized for several years. In 1980, Makinodan [12] conceptualized the term immunosenescence, while in 2000, Franceschi et al. [13] highlighted that aging is marked by a progressive increase in pro-inflammatory status, known as inflamm-aging. This persistent systemic inflammation is associated with cellular senescence, immune decline, organ dysfunction, and age-related diseases. Simultaneously, chronic inflammation accelerates immunosenescence, resulting in reduced immune capacity and a weakened ability to clear senescent cells and inflammatory agents, thus perpetuating a detrimental cycle [14].

Therefore, this study aimed to determine whether extreme longevity is associated with better control of IMFLAM responses compared to older individuals, including nonagenarians and younger centenarians. To achieve this, we measured the INFLA-score, which assesses the synergistic effects of inflammatory markers [15], and the Systemic Inflammation Response Index (SIRI), a composite marker derived from peripheral blood cell counts that reflects the immune-inflammatory balance [16]. We also analyzed the Aging-Related Immune Phenotype (ARIP) indicators, which provide a more comprehensive understanding of how T-cell immunity relates to health compared to individual T-cell subsets [17]. The results obtained support the notion that effective control of IMFLAM response can promote extreme longevity.

## 2. Materials and Methods

### 2.1. Study Population

In this study, the dataset used for score calculation, along with the raw data, was collected as part of the project “Discovery of Molecular and Genetic/Epigenetic Signatures Underlying Resistance to Age-Related Diseases and Comorbidities” (DESIGN, 20157ATSLF, funded by the Italian Ministry of Education, University, and Research), from June 2017 to March 2023. The study design complies with the Declaration of Helsinki and its amendments and is approved by the Ethics Committee of Palermo University Hospital (Nutrition and Longevity, No. 032017).

We selected healthy participants, accounting for age-related physiological decline in organs and systems, such as hearing and vision impairments, and included only those with a maximum of one debilitating condition. We also included cognitively capable individuals, though not necessarily fully unimpaired. Those with chronic debilitating conditions like neoplastic or autoimmune disorders, acute diseases such as infections, or severe dementia were excluded. Individuals who had taken immunomodulatory drugs within the past six months were also not eligible. Recruitment for adults (<65 years) and older adults (<90 years) occurred at the University of Palermo through social media and word-of-mouth referrals, while LLIs (<105 years) and the oldest centenarians (>105 years) were recruited at home due to difficulties in clinic attendance. Many of our LLIs and oldest centenarians lived in small villages with family support, often lacked independent transportation, and required a caregiver. For this group, we obtained lists of the oldest residents from municipalities or general practitioners and contacted families by phone to confirm interest and ensure inclusion and exclusion criteria were met. A team of demographers, biologists, and physicians from the University of Palermo conducted a detailed questionnaire to collect demographic, clinical, and medical history information, as well as functional and cognitive data from participants [11,18].

Since the purpose of our study was to determine whether extreme longevity is associated with better control of IMFLAM responses, we enriched our sample with centenarians, particularly those at the oldest ages. Since more than 80% of Italian centenarians are women, a figure that rises to 90% for those aged 105 and above [19], this led to a disproportion between the number of women and men in the oldest age groups (see below).

For the inflammatory analysis, a total of 249 participants (139 females; 110 males) aged between 19 and 111 years were included, with the exception of the INFLA-score, which had two missing values (N = 247, 1 female LLI and 1 female supercentenarian). To better understand trends across each age range, we conducted comparative analyses by dividing the entire cohort into four age groups: adults (45 males, 46 females; age range: 19.5–64.7 years), older adults (40 males, 36 females; age range: 65.0–89.3 years), LLIs (24 males, 45 females; age range: 90.5–104.7 years), and semi- and supercentenarians (1 male, 12 females; age range: 105.4–111.8 years). For the immunological evaluation, a subset of 54 participants (28 females; 26 males) aged between 19 and 110 years was randomly selected from the full group, with consideration given to sex and age. The four groups were composed as follows: adults (10 males, 10 females; age range: 19.5–63.60 years); older adults (8 males, 7 females; age range: 68.5–87.3 years); LLIs (7 males, 4 females; age range: 93.3–104.7 years); and semi- and supercentenarians (1 male, 7 females; age range: 105.7–110.3 years).

### 2.2. Age Validation of Semi- and Supercentenarians

For all recruited centenarians’ age validation, the information on their identity cards and tax codes, along with those of their offspring caregivers, was cross-verified for consistency with each other and with reported marriage dates and other family records. The identity cards of semi-supercentenarians and supercentenarians are validated through the Italian Institute of Statistics (ISTAT) semi-supercentenarians survey [19]. This survey gathers data on residents, both living and deceased, aged 105 and older. The primary data source for validation is the National Register of the Resident Population. All municipalities with at least one semi-supercentenarian or supercentenarian resident submit a birth or death certificate and additional demographic details. For individuals still alive at age 105, an annual follow-up is conducted until their death, which is then recorded in the database. As long as the individual remains alive, the validation process is continuous, with ISTAT “re-validating” previous data each year. Additionally, we identified and recruited centenarians aged 108+ through the website “Supercentenari d’Italia” [20]. For age validation, the manager of the website requires three additional documents beyond the Identity Card and Tax Code: an original birth or baptism certificate, a certificate of existence from an intermediate age (anytime between 20 and 100 years), and, for supercentenarians, an additional certificate of existence.

### 2.3. Inflammatory Scores: INFLA-Score and SIRI

The INFLA-score was calculated by creating deciles for leukocyte (WBC) count, neutrophil to lymphocyte ratio (NLR) values, platelet count, and C reactive protein (CRP). Each biomarker’s data were split into ten groups according to value, with the first decile representing the lowest values, the second decile representing slightly higher values, continuing in this way until the tenth decile, which included the highest values. These biomarker deciles were assigned scores ranging from low values (−4 to −1) to high values (+1 to +4), while intermediate values were assigned a score of 0. The INFLA-score is obtained by summing the scores of the four components, resulting in a possible range from −16 to +16 [15]. The SIRI was calculated according to Qi et al. (neutrophils x monocytes/lymphocytes) [16]. Leukocyte and inflammatory values were obtained as previously described [11,18].

### 2.4. Immunological Scores: ARIP

The ARIP includes the CD4/CD8 ratio, CD4^+^ and CD8^+^ T Naïve cells, and the T Naïve (T_N_)/T_M_ (T_M_ = T Central Memory (T_CM_) + T Effector Memory (T_EM_) + Terminally Differentiated Effector Memory (T_EMRA_)) for both CD4^+^ and CD8^+^ T cells [17], calculated on a subcohort of 54 individuals [11].

### 2.5. Statistics

The correlation between the various parameters and age was examined using simple linear regression analysis. A one-way ANOVA test was used to compare data between the different age groups, applying Tukey’s correction. For all statistical analyses, only *p*-values ≤ 0.05 were considered significant.

## 3. Results

### 3.1. INFLA-Score

Figure S1a,c,e,g shows the correlation between age and WBC count, NLR values, platelet count, CRP, and Figure S1b,d,f,h shows their comparisons between the different age groups. It is possible to note a statistically significant correlation between age and NLR (Figure S1c; R^2^= 0.066; *p*-value <0.0001) and between age and CRP (Figure S1g; R^2^= 0.064; *p*-value <0.0001). Conversely, platelet values seemed to decrease with age, and this change was statistically significant (Figure S1e; R^2^ = 0.036; *p*-value = 0.003). The comparison between age groups showed statistically significant differences, except for WBC count (Figure S1b). Statistically significant results were observed in the comparison between age groups for NLR, platelet count, and CRP (Figure S1d,f,h). For NLR, the values for adults and older individuals were significantly lower than those for LLIs (Figure S1d; *p*-values = 0.002 and 0.043, respectively) and for semi- and supercentenarians (Figure S1d; Table S1; *p*-values = 0.004 and 0.019, respectively). For platelet count, the only significant differences were observed between adults and LLIs (Figure S1f; Table S1; *p*-value = 0.044). For CRP (Figure S1h; Table S1), the values for adults and older individuals were significantly lower than those for LLIs (*p*-values <0.0001 and 0.009, respectively), while the values for semi- and supercentenarians were significantly lower than those for LLIs (*p*-value = 0.026).

**Figure 1:**
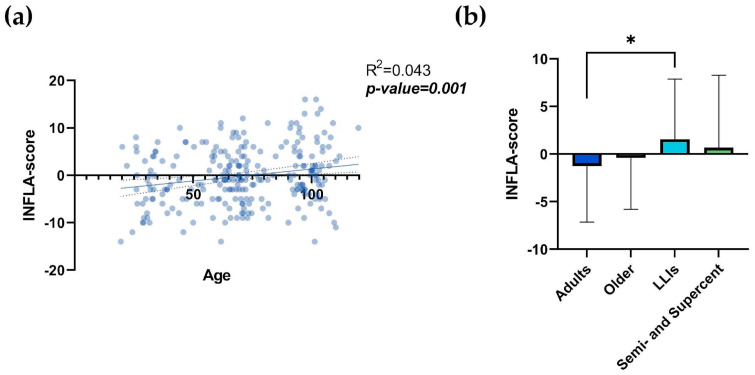
INFLA-score and age. Linear regression analysis shows the relationship between INFLA-score values and age (**a**) in N = 247 individuals. Each point represents data from a healthy donor. The dashed line represents the 95% confidence interval, while the solid line indicates the data trend. Column bar graphs show differences between the mean of the values of INFLA-score (**b**) from each age group obtained by one-way ANOVA test. The standard deviation (SD) and *p*-values are shown on the graphs. The vertical lines with horizontal caps represent the mean ± SD. Statistical significance between groups in the columns is denoted by horizontal lines above the bars, marked with asterisks (*). The number of “*” indicates the level of significance: * *p*-value ≤0.05; LLIs: Long-Lived Individuals; Semi- and Supercent: Semi- and Supercentenarians; R^2^: R squared; ns: not significant; *p*-value: statistical significance.

Figure 1a clearly shows an increase in the INFLA-score with age (R^2^ = 0.043; *p*-value = 0.001), with a value range between −14 and +16, and a median value of −1. Notably, there is meaningful heterogeneity, especially at extreme ages (Figure S2). Analyzing the correlation with age by sex (Figure S3a), significance was retained only for males (*p*-value = 0.0005). A comparison of scores across age groups (Figure 1b; Table 1) revealed a statistically significant difference between the adult and LLIs groups (*p*-value = 0.018). No significant differences were observed between the semi- and supercentenarian groups and the other groups (Figure 1b; Table 1). Scores by age group were not analyzed by sex due to the unequal representation of the two sexes among the oldest centenarians (65% of LLIs and 92% of semi- and supercentenarians were women).

### 3.2. SIRI Analysis

Regarding the SIRI analysis, Figure S4a,c,e show the correlation between age and leukocyte populations, and Figure S4b,d,f show the leukocyte count in the different age groups. It is possible to note a positive and statistically significant correlation between age and monocyte count (Figure S4c; R^2^ = 0.039; *p*-value = 0.002), and conversely, a negative correlation between age and lymphocytes (Figure S4e; R^2^ = 0.017; *p*-value = 0.042). The comparison between age groups did not show statistically significant differences for any of the leukocyte populations considered, except for monocyte values between the adult and LLIs groups (Figure S4d; Table S2; *p*-value = 0.011).

**Figure 2:**
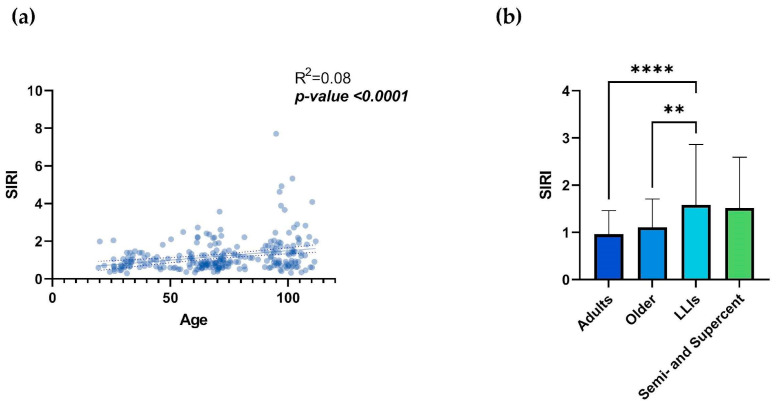
SIRI and age. Linear regression analysis shows the relationship between SIRI values and age (**a**) in N = 249 individuals. Each point represents data from an individual healthy donor. The dashed line represents the 95% confidence interval, while the solid line indicates the data trend. Column bar graphs show differences between the mean of the values of SIRI (**b**) from each age group obtained by one-way ANOVA test. The SD and *p*-values are shown on the graphs. The vertical lines with horizontal caps represent the mean ± SD. Statistical significance between groups in the columns is denoted by horizontal lines above the bars, marked with asterisks (*). The number of “*” indicates the level of significance: ** *p*-value ≤0.01; **** *p*-value ≤0.0001; SIRI = Systemic Inflammation Response Index; LLIs = Long-Lived Individuals; Semi- and Supercent: Semi- and Supercentenarians; R^2^: R squared; ns: not significant. *p*-value: statistical significance.

However, considering SIRI, simple linear regression shows a highly significant statistical correlation of this index with age, with an increase in SIRI values according to age (Figure 2a; R^2^ = 0.08; *p*-value <0.0001), although significant heterogeneity exists at extreme ages (Figure S5). Analyzing the correlation with age by sex (Figure S3b), significance was maintained for both females (*p*-value = 0.004) and males (*p*-value = 0.0002). Comparing the index between age groups, there was a statistically significant difference between the adult and LLIs groups (*p*-value <0.0001) and between the older and LLIs groups (*p*-value = 0.005) (Figure 2b; Table 1). Interestingly, no significant differences were observed between the semi- and supercentenarians and the other groups (Figure 2b; Table 1). As for the INFLA-score, the indices were not analyzed by sex across age groups due to the disproportionate representation of the two sexes among the oldest centenarians.

### 3.3. ARIP Indicators

According to Ramasubramanian et al. [17], ARIP is represented by CD4^+^ and CD8^+^ Naïve T cells, the CD4/CD8 ratio, and the CD4^+^ TN/TM and CD8^+^ TN/TM ratios. Figure S6a shows a significant decrease in CD4^+^ TN with age (R^2^ = 0.094; *p*-value = 0.024) and a significant increase in CD4^+^ TM (R^2^ = 0.095; *p*-value = 0.024). However, the comparison between age groups did not show statistically significant differences between the groups (Figure S6b,c). Figure S6d shows a significant decrease in CD8^+^ TN with age (R^2^ = 0.24; *p*-value = 0.0002) and a significant increase in CD8^+^ TM (R^2^ = 0.24; *p*-value = 0.0002). The comparison between age groups for CD8^+^ TN and TM showed statistically significant differences (Figure S6e,f). Adult values of CD8^+^ TN were significantly higher than those of older individuals (*p*-value = 0.018), LLIs (*p*-value = 0.014), and semi- and supercentenarians (Figure S6e; Table S3; *p*-value = 0.013). Adult values of CD8^+^ TM were significantly lower than those of older individuals (*p*-value = 0.026), LLIs (*p*-value = 0.015), and semi- and supercentenarians (Figure S6f; Table S3; *p*-value = 0.013). Previously, we demonstrated in this sample of subjects that there was no significant correlation between the CD4/CD8 ratio and age [11].

**Figure 3:**
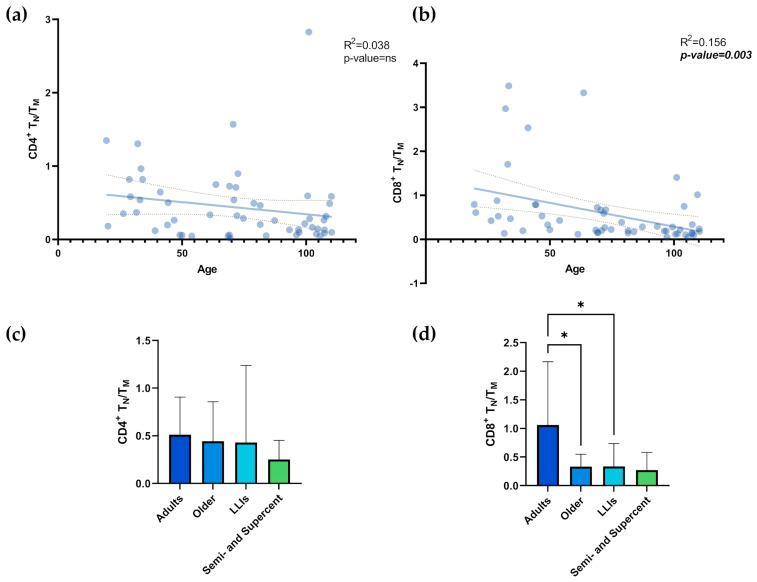
T_N_/T_M_ values and age. Linear regression analysis shows the relationship between CD4^+^ T_N_/T_M_ values (**a**), CD8^+^ T_N_/T_M_ values (**b**), and age in N = 54 individuals. Each point represents data from an individual healthy donor. The dashed line represents the 95% confidence interval, while the solid line indicates the data trend. Column bar graphs show differences between the mean of the values of CD4^+^ T_N_/T_M_ values (**c**), CD8^+^ T_N_/T_M_ values (**d**) from each age group obtained by one-way ANOVA test. The SD and *p*-values are shown on the graphs. The vertical lines with horizontal caps represent the mean ± SD. Statistical significance between groups in the columns is denoted by horizontal lines above the bars, marked with asterisks (*). The number of “*” indicates the level of significance: * *p*-value ≤0.05; T_N_: T Naïve (CD45RA^+^CD27^+^); T_M_:T_CM_ (CD45RA^−^CD27^+^) + T_EM_ (CD45RA^−^CD27^−^) + T_EMRA_ (CD45RA^+^CD27^−^); LLIs: Long-Lived Individuals; Semi- and Supercent: Semi- and Supercentenarians; R^2^: R squared; ns: not significant; *p*-value: statistical significance.

For the TN/TM cell ratio, no significant age-related correlation was found for the CD4^+^ TN/TM ratio (Figure 3a; R^2^ = 0.038; *p*-value = ns). In contrast, a significant correlation with age was found for the CD8^+^ TN/TM ratio (Figure 3b; R^2^ = 0.156; *p*-value = 0.003). Accordingly, the comparison between age groups did not show statistically significant differences for the CD4^+^ TN/TM ratio (Figure 3c), whereas, for the CD8^+^ TN/TM ratio (Figure 3d), the values for adults were significantly higher than those of older individuals and LLIs (Table 1; *p*-values = 0.024 and 0.048, respectively). Intriguingly, no significant differences were observed between the semi- and supercentenarians and the other groups (Figure 3d).

## 4. Discussion

Inflamm-aging, that is, the chronic low-grade inflammation that occurs during aging, is characterized by increased systemic levels of pro-inflammatory mediators, resulting from a complex interaction of biological, genetic, and environmental factors. It has a significant impact on multiple body systems, contributing to the onset and progression of numerous age-related diseases and the general decline of health in older people. In addition, the inflammatory process is favored by the decline of the immune system and, in turn, aggravates immune aging [13,21,22,23,24,25,26,27,28]. For all these reasons, the identification of good markers to evaluate the systemic inflammatory status is crucial. Since sometimes the single parameter does not represent a good evaluation index of the effective systemic condition, the possibility of calculating scores, by putting together multiple markers, is more informative, ignoring the variability presented by differences in units, mean intakes, and biological actions given by the analysis of individual biomarkers.

The INFLA-score and SIRI, two composite indices summarizing the effect of multiple inflammatory biomarkers, were calculated to measure the level of chronic inflammation [15,16]. It is noteworthy that the underlying parameters did not always show overlapping significance. Thus, integrating various inflammatory biomarkers, the INFLA-score captures the complexity of inflammation as a multifaceted process involving both plasmatic and cellular components [15]. It also provides a useful tool for large-scale epidemiological studies, summarizing the variability of inflammation among different individuals [15,29,30,31,32].

SIRI is a reliable prognostic marker in various diseases, helping to predict patient outcomes, survival rates, and disease progression [16]. It is obtained by integrating different aspects of the inflammatory response through the combination of neutrophil, monocyte, and lymphocyte counts. In this way, SIRI provides a more comprehensive view of systemic inflammation compared to single markers, offering several advantages in both clinical and research settings [16,33,34,35,36].

The results obtained in the present study for the two inflammatory indices, INFLA-score and SIRI, clearly demonstrate an age-related increase in the pro-inflammatory state, with the exception of semi- and supercentenarians, whose values are not significantly different from those of other groups. These findings are consistent with the well-known age-related increase in the pro-inflammatory state [13,24,25,27] and support the idea that controlling inflammatory responses may contribute to promoting longevity, especially extreme longevity, as a higher pro-inflammatory status is an independent factor for mortality [5,11,27,37,38,39,40,41]. It is also noteworthy that the increase in indices is lower in women: for the INFLA-score, the female correlation with age is not significant, while for SIRI, it is significant but with a lower *p*-value than that for men. These findings align with the known lower baseline level of inflammation in women compared to men, as older men show higher monocyte activity and inflammatory responses than older women. These characteristics may contribute to the longer lifespan observed in women compared to men [27,42,43,44].

The mechanisms proposed to explain the lower inflammatory values in semi- and supercentenarians, compared to LLIs, include factors such as a healthy lifestyle during youth, characterized by an anti-inflammatory Mediterranean diet and regular physical activity, anti-inflammatory genetic polymorphisms, residence in small towns with low levels of pro-inflammatory pollution, and robust family support networks that help mitigate stress. Additionally, the effective control of senescent cells, which are known to produce pro-inflammatory mediators, may also contribute to this outcome [5,18,27,45].

As it is known, not only do various immunological parameters change throughout life [27], but certain age-related changes have also been associated with an increased risk of mortality [17,41,46]. The alterations in the adaptive immune system during the aging process contribute to an ARIP adaptation [17]. A recent study analyzed the associations of ARIP measures with chronological age, biological age, and multimorbidity outcomes in a large sample of Americans over 55 years old [17]. However, the sample included a negligible percentage of centenarians and no semi- or supercentenarians (age (mean  ±  SE) 68.65  ±  0.26) [17]. The relationships between rising multimorbidity, mortality, and biological age suggested that CD4^+^ T_N_/T_M_ and CD4^+^ T Naïve cells might serve as biomarkers for detecting individuals at greater risk of accelerated aging and related morbidities, as well as increased mortality [17]. This is supported by findings from the Multi-Ethnic Study of Atherosclerosis, which show that Naïve and memory CD4^+^ T cells are linked to type II diabetes and subclinical atherosclerosis in a cross-sectional manner [47,48]. The CD8^+^ T_N_/T_M_ ratio was then associated with a lower risk of cancer, heart disease, and diabetes, and it was predictive of successful aging, as well as CD8^+^ T Naïve. Regarding another ARIP parameter, the CD4/CD8 ratio, it does not condition the rise of age-related diseases [17].

The only significant differences found in our work, regarding the comparison between the various groups, concerned the CD8^+^ T cells. However, in this study sample, multiple regression analysis showed that levels of CD8^+^ T Naïve, T_EM_, and T_EMRA_ cells are more strongly correlated with the degree of cytomegalovirus seropositivity than with age (unpublished observations). Despite these reservations about the significance of the data, it is interesting to note that there are no significant differences in the values of the CD8^+^ T_N_/T_M_ ratio between the groups of younger individuals and the semi- and supercentenarians, with a great heterogeneity of values. In contrast, the data for older adults and LLIs were significantly different from those of younger adults, explaining the observed significant age-related correlation. As previously noted, this datum could be considered, on average, as an indicator of successful aging, at least in semi- and supercentenarians. On the other hand, recent studies of lymphocyte subsets in semi- and supercentenarians suggest that immune system aging changes should be considered as a specific adaptation that enables the oldest centenarians to successfully cope with a lifetime of antigenic challenges and achieve extreme longevity [11,49,50].

Our study has several limitations. The first limitation is the cross-sectional nature of the data. Cross-sectional studies are useful for assessing prevalence and identifying potential associations between variables, but they cannot provide definitive answers regarding causality or temporal changes. Furthermore, the number of enrolled oldest centenarians was relatively small. However, it is important to note that semi- and supercentenarians are relatively rare (the ratio of supercentenarians to centenarians is 1 in 1000) [5,9]. The sex distribution was imbalanced, as it reflects the female-to-male ratio among Italian centenarians, which is 85% to 15% [19]. Moreover, according to the Supercentenarians of Italy website, as of June 2024, there were 29 living supercentenarians (aged over 110), only one of whom was male, and 205 semi-supercentenarians (aged over 107), of whom only 15 were men, resulting in a ratio of nearly 13 women for every man [20]. Therefore, caution should be exercised when interpreting the data from the sex-based analysis. Finally, in our analysis, we aimed to use standardized methods to assess the overall inflammatory state, minimizing the variability of individual inflammatory markers. Consequently, we did not take into consideration the analysis of chemokines and other specific markers, belonging to the inflammatory clock (e.g., CXCL9), known to play a role in the inflammatory process in older people [51].

To address most of the limitations discussed in the previous paragraph, future directions for our work should include the involvement of additional national centers interested in studying semi- and supercentenarians to obtain a larger sample size, particularly of men. This expansion would also allow for the inclusion of analyses on chemokines [51] and cytokines [52,53] that are known to be positively or negatively involved in inflamm-aging but have not yet been studied in the oldest centenarians.

## 5. Conclusions

Our results extend and reinforce the idea that controlling IMFLAM responses plays a significant role in achieving extreme longevity. This does not exclude the involvement of other organs and systems. The innate immune system (inflammation) and the adaptive immune system (lymphocytes and antibodies) have received more extensive research attention compared to other systems, especially given their suitability for ex vivo studies. Additionally, it is worth considering that an efficient immune system could be the effect of a well-functioning organism rather than its cause. Finally, the heterogeneity observed in the values for semi- and supercentenarians should not be surprising, because as evolutionary medicine teaches us about disease, there is no single model of aging: everyone ages in a unique way because their genotypes and exposomes are unique.

## Figures and Tables

**Table 1:** Immune-Inflammatory indexes and scores. Mean ± SD of immune-inflammatory indexes and scores according to age groups.

**Variable**	**Adults** **(N = 91)**	**Older** **(N = 76)**	**LLIs** **(N = 69)**	**Semi- and Supercentenarians** **(N = 13)**	**Significant Comparisons**	***p*-Value**
INFLA-score ^1^	−1.253 ± 5.891	−0.408 ± 5.394	1.559 ± 6.316	0.667 ± 7.608	Adults vs. LLIs	=0.018
SIRI	0.963 ± 0.5	1.107 ± 0.6	1.583 ± 1.28	1.519 ± 1.073	Adults vs. LLIs	<0.0001
					Older vs. LLIs	=0.005
**Variable**	**Adults** **(N = 20)**	**Older** **(N = 15)**	**LLIs** **(N = 11)**	**Semi- and Supercentenarians** **(N = 8)**	**Significant comparisons**	***p*-Value**
CD4^+^ T_N_/T_M_	0.512 ± 0.393	0.443 ± 0.415	0.429 ± 0.809	0.252 ± 0.2	None	Ns
CD8^+^ T_N_/T_M_	1.061 ± 1.104	0.335 ± 0.215	0.337 ± 0.401	0.272 ± 0.312	Adults vs. Older	=0.024
					Adults vs. LLIs	=0.048

^1^ The data from two individuals were missing, so 247 individuals, instead of 249, have been considered for the calculation. SD: Standard Deviation; ns: not significant; SIRI: Systemic Inflammation Response Index; LLIs: Long-Lived Individuals; T_N_: T Naïve (CD45RA^+^CD27^+^); T_M_: TCM (CD45RA^−^CD27^+^) + TEM (CD45RA^−^CD27^−^) + TEMRA (CD45RA+CD27−). *p*-values obtained from the one-way ANOVA test are reported.

## Data Availability

The data that support the findings of this study are available from the corresponding author, upon reasonable request.

## Supplementary Materials

The following supporting information can be downloaded at: https://www.mdpi.com/article/10.3390/biology13121010/s1, Supplementary Figures and Tables: INFLA-score computation; Figure S1: The parameters of the INFLA-score; Figure S2: Semi- and Supercentenarians INFLA-score; Figure S3: The parameters of SIRI analysis; Figure S4: Semi- and Supercentenarians SIRI; Figure S5: The parameters of T_N_/T_M_; Figure S6: Correlation with age by sex; Table S1: INFLA-score parameters; Table S2: SIRI parameters; Table S3: TN-TM parameters.
