# Supplementary material for: Immune-Inflammatory Response in Lifespan—What Role Does It Play in Extreme Longevity? A Sicilian Semi- and Supercentenarians Study
Source: Biology (Basel). 2024 Dec 4;13(12):1010. doi: 10.3390/biology13121010 (PMC11673833; doi:10.3390/biology13121010)
Supplement: Supplementary file 1 [file biology-13-01010-s001.zip › biology-3252578-supplementary.pdf]

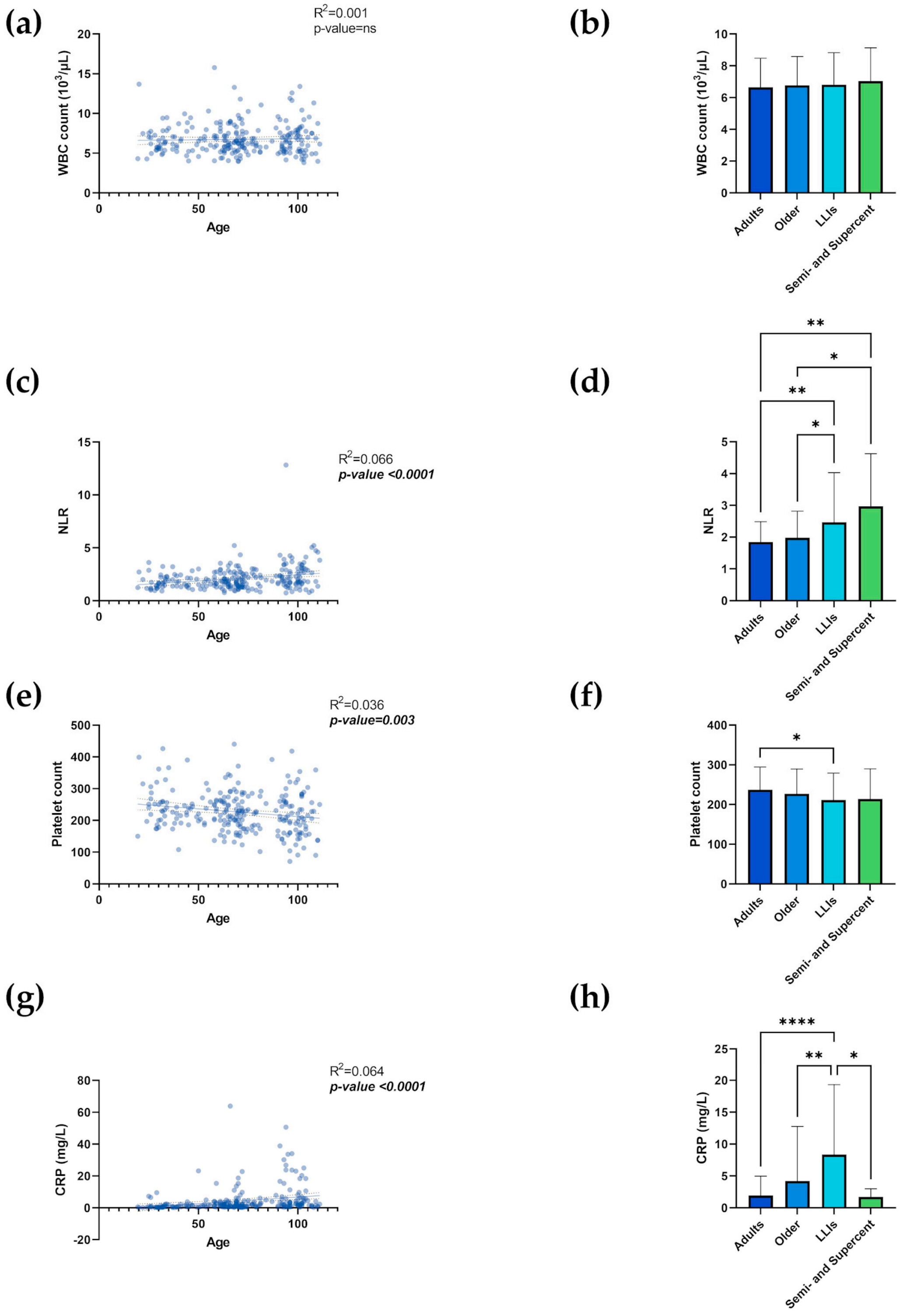

Figure S1. The parameters of the INFLA-score. Linear regression analysis shows the relationship between White Blood Cells (WBC) (a), Neutrophil-to-Lymphocyte Ratio (NLR) (c), Platelet count (e), C-Reactive Protein (CRP) (g)and age in N = 247 individuals. Each point represents data from a healthy donor. The dashed line represents the 95% confidence interval, while the solid line indicates the data trend. Column bar graphs show differences between the mean of the values of WBC count (b), NLR (d), Platelet count (f), and CRP (h) from each age group obtained by one-way ANOVA test. The standard deviation (SD) and *p*-values are shown on the graphs. The vertical lines with horizontal caps represent the mean ± SD. Statistical significance between groups in the columns is denoted by horizontal lines above the bars, marked with asterisks (*). The number of "*" indicates the level of significance: **p*-value ≤0.05; ***p*-value ≤0.01; *****p*-value ≤0.0001. LLIs: Long-Lived Individuals; Semi and Supercent: Semi- and Supercentenarians; R^2: R squared; ns: not significant. *p-value*: statistical significance.

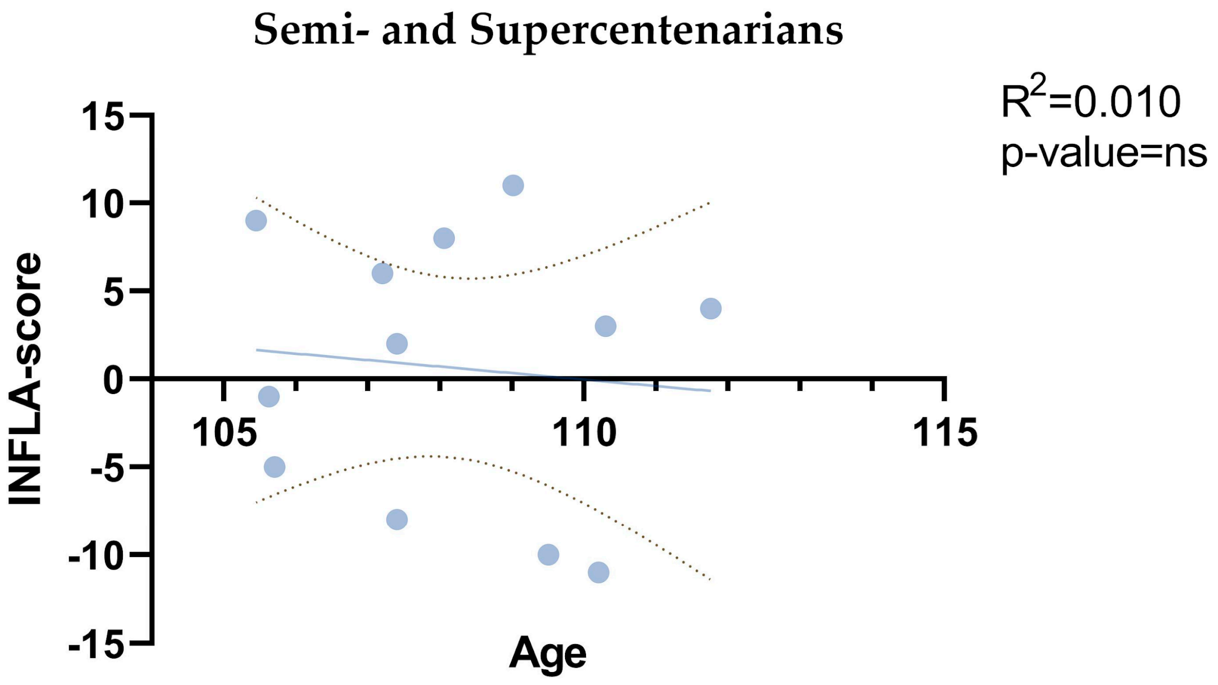

Figure S2. Semi- and Supercentenarians INFLA-score. Linear regression analysis shows the relationship between INFLA-score and age in the age group of semi- and supercentenarians (N = 12). Each point represents data from a healthy donor. The coefficient of determination and p-values are shown on the graphs. The dashed line represents the 95% confidence interval, while the solid line indicates the data trend. R^2: R squared; ns: not significant. *p-value*: statistical significance.

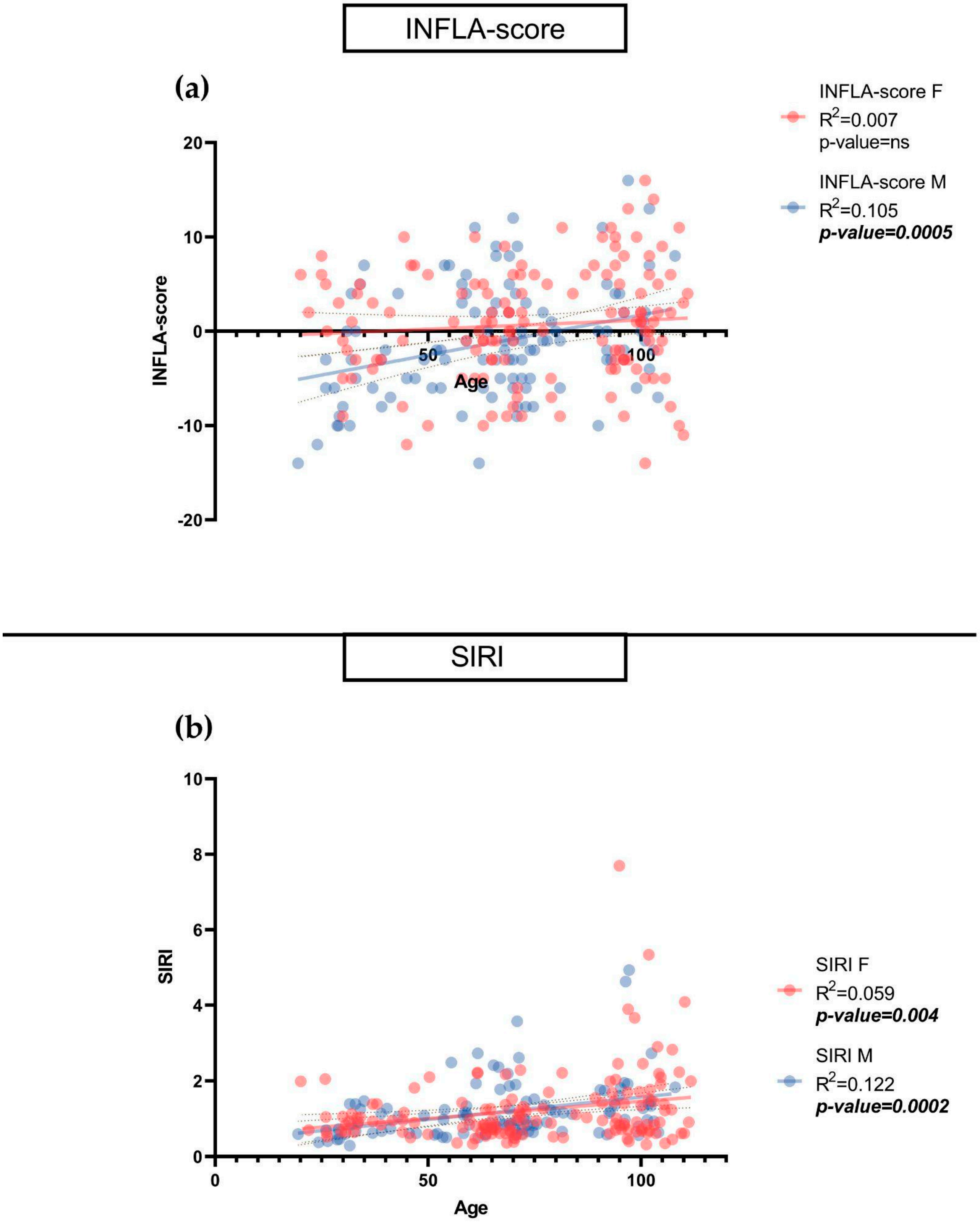

Figure S3. Correlation with age by sex (a) INFLA-score. Linear regression analysis shows the relationship between INFLA-score values and age (a) in 137 females and 110 males. (b) SIRI. Linear regression analysis shows the relationship between SIRI values and age (a) in 139 females and 110 males. Each point represents data from an individual healthy donor. The dashed line represents the 95% confidence interval, while the solid line indicates the data trend. SIRI: Systemic Inflammation Response Index; R^2: R squared; ns:not significant. *p-value*: statistical significance.

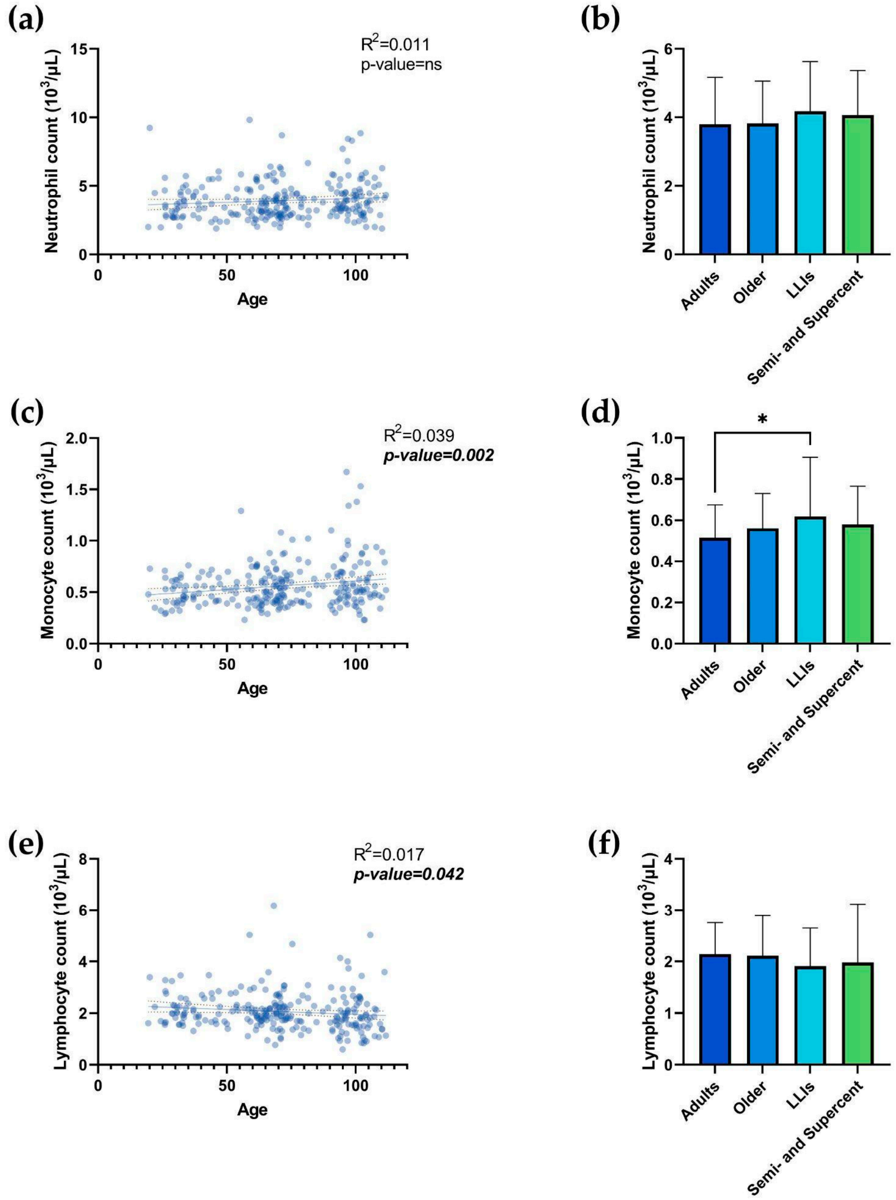

Figure S4. The parameters of SIRI analysis. Linear regression analysis shows the relationship between Neutrophil count (a), Monocyte count (c), Lymphocyte count (e), and age in N = 249 individuals. Each point represents data from an individual healthy donor. The dashed line represents the 95% confidence interval, while the solid line indicates the data trend. Column bar graphs show differences between the mean of the values of Neutrophil count (b), Monocyte count (d), and Lymphocyte count (f), from each aged group obtained by one-way ANOVA test. The SD and *p*-values are shown on the graphs. The vertical lines with horizontal caps represent the mean ± SD. Statistical significance between groups in the columns is denoted by horizontal lines above the bars, marked with asterisks (*). The number of "*" indicates the level of significance: *p-value ≤0.05; SIRI: Systemic Inflammation Response Index; LLIs: Long-Lived Individuals; Semi and Supercent: Semi- and Supercentenarians; R^2: R squared; ns: not significant. *p-value*: statistical significance.

Semi- and Supercentenarians

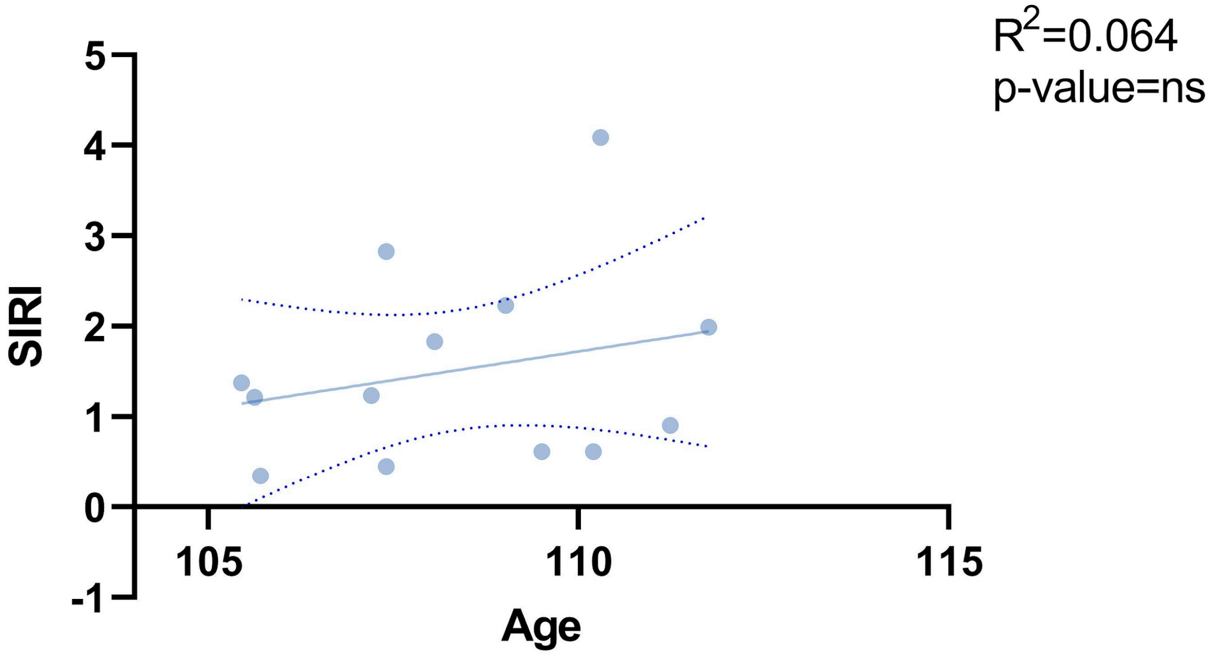

Figure S5. Semi- and Supercentenarians SIRI. Linear regression analysis shows the relationship between SIRI and age in the age group of semi- and supercentenarians (N = 13). Each point represents data from a healthy donor. The coefficient of determination and p-values are shown on the graphs. The dashed line represents the 95% confidence interval, while the solid line indicates the data trend. SIRI: Systemic Inflammation Response Index; R^2: R squared; ns: not significant. *p-value*: statistical significance.

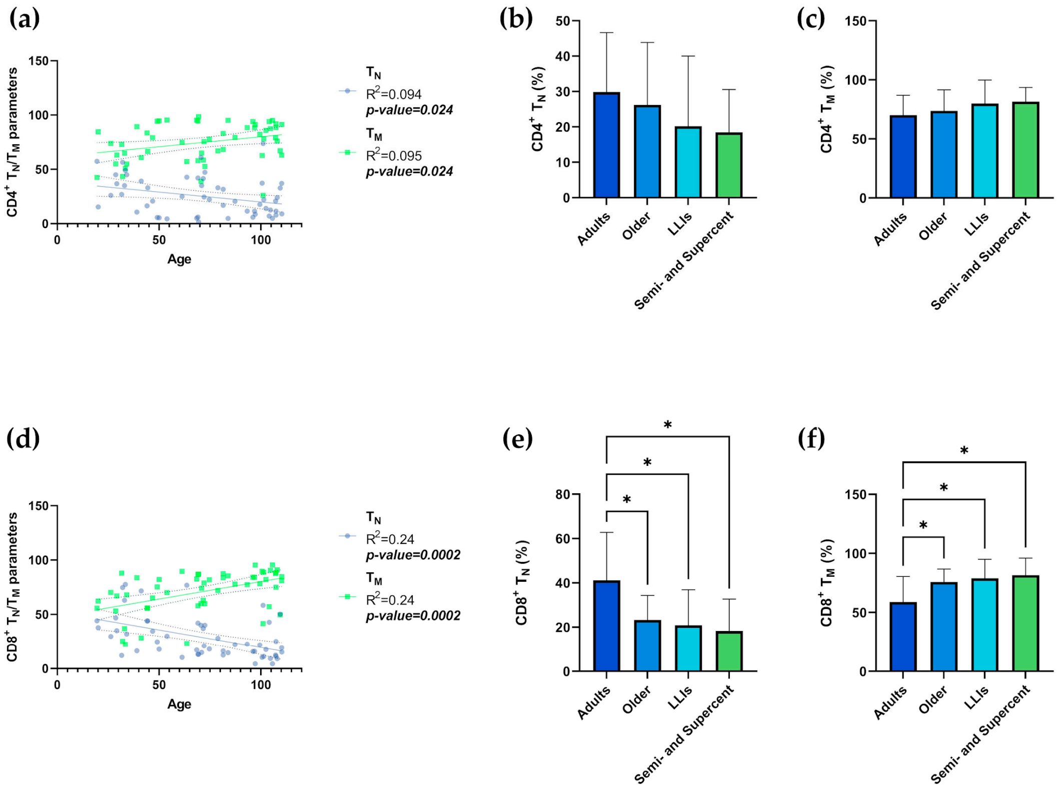

Figure S6. The parameters of T_N/T_M. Linear regression analysis shows the relationship between CD4+ T_N (blue line), CD4+ T_M (green line) (a) and CD8+ T_N (blue line), CD8+ T_M (green line) (d) and age in N= 54 individuals. Each point represents data from a healthy donor. The dashed line represents the 95% confidence interval, while the solid line indicates the data trend. Column bar graphs show differences between the mean of the values of CD4+ T_N (b), CD4+ T_M (c), CD8+ T_N (e), CD8+ T_M (f) from each aged group obtained by one-way ANOVA test. The SD and p-values are shown on the graphs. The vertical lines with horizontal caps represent the mean ± SD. Statistical significance between groups in the columns is denoted by horizontal lines above the bars, marked with asterisks (*). The number of "*" indicates the level of significance: **p*-value ≤0.05; T_N: T Naïve (CD45RA+CD27+); T_M: T_{CM} (CD45RA-CD27+) + T_{EM} (CD45RA-CD27-) + T_{EMRA} (CD45RA+CD27-); LLIs: Long-Lived Individuals; Semi and Supercent: Semi- and Supercentenarians; R^2 = R squared; ns: not significant; *p-value*: statistical significance.

Table S1. INFLA-score parameters. Mean ± SD of INFLA-score parameters according to age groups.

Variable	Adults (N= 91)	Older (N= 76)	LLIs (N= 68)	Semi- and Supercentenarians (N= 12)	Significant comparisons	p-Value
WBC count ($10^3/\mu L$)	6.65±1.83	6.77±1.82	6.81±2.02	7.03±2.10	None	Ns
NLR	1.85±0.64	1.98±0.84	2.47±1.56	2.97±1.66	Adults vs. LLIs<br>Adults vs. Semi- and Supercentenarians<br>Older vs. LLIs<br>Older vs. Semi- and Supercentenarians	=0.002<br>=0.004<br>=0.043<br>=0.019
Platelet count	237.4±57.26	227±62.33	211.4±67.62	214.1±75.68	Adults vs. LLIs	=0.044
CRP (mg/L)	1.94±3.03	4.23±8.53	8.36±10.96	1.69±1.31	Adults vs. LLIs<br>Older vs. LLIs<br>LLIs vs. Semi- and Supercentenarians	<0.0001<br>=0.009<br>=0.026

SD: Standard Deviation; ns: not significant; LLIs: Long-Lived Individuals; WBC White Blood Cells; NLR: Neutrophil-to-Lymphocyte Ratio; CRP: C-Reactive Protein. p-values obtained from the one-way ANOVA test are reported

Table S2. SIRI parameters. Mean ± SD of SIRI parameters according to age groups.

Variable	Adults (N= 91)	Older (N= 76)	LLIs (N= 69)	Semi- and Supercentenarians (N= 13)	Significant comparisons	p-value
Neutrophil count (10^3/μL)	3.8±1.37	3.82±1.24	4.18±1.45	4.07±1.30	None	ns
Monocyte count (10^3/μL)	0.52±0.16	0.56±0.17	0.62±0.29	0.58±0.19	Adults vs. LLIs	0.011
Lymphocyte count (10^3/μL)	2.14±0.61	2.11±0.78	1.91±0.74	1.98±1.13	None	ns

SIRI: Systemic Inflammation Response Index; SD: Standard Deviation; ns: not significant; LLIs: Long-Lived Individuals. p-values obtained from the one-way ANOVA test are reported

Table S3. T_N-T_M parameters. Mean ±S D of $CD4^+$ and $CD8^+$ T_N-T_M according to age groups.

Variable	Adults (N= 20)	Older (N= 15)	LLIs (N= 11)	Semi- and Supercentenarians (N= 8)	Significant comparisons	p-value
			$CD4^+$ T_N-T_M parameters			
$CD4^+$ T_N	29.83±16.79	26.21±17.66	20.21±19.82	19.49±12.6	None	ns
$CD4^+$ T_M	70.14±16.80	73.85±17.70	79.90±81.53	81.53±12.09	None	ns
			$CD8^+$ T_N-T_M parameters			
$CD8^+$ T_N	41.16±21.62	23.28±11.09	20.91±16.01	18.31±14.36	Adults vs. Older<br>Adults vs. LLIs<br>Adults vs. Semi- and Supercentenarians	=0.018<br>=0.014<br>=0.013
$CD8^+$ T_M	58.95±21.67	75.97±10.83	79.07±16	81.70±14.36	Adults vs. Older<br>Adults vs. LLIs<br>Adults vs. Semi- and Supercentenarians	=0.026<br>=0.015<br>=0.013

*SD: Standard Deviation; ns: not significative; LLIs: Long-Lived Individuals; T_N: T Naïve ($CD45RA^+CD27^+$); T_M: T_{CM} ($CD45RA^-CD27^+$) + T_{EM} ($CD45RA^-CD27^-$) + T_{EMRA} ($CD45RA^+CD27^-$). p-values obtained from the one-way ANOVA test are reported.
